# Pain perception and dental anxiety during periodontal probing in patients visiting community oral health programme: a cross sectional study

**DOI:** 10.1186/s12903-021-01437-y

**Published:** 2021-02-23

**Authors:** Abanish Singh, Ashish Shrestha, Tarakant Bhagat

**Affiliations:** 1 Narayani Hospital, Birgunj, Parsa, Nepal; 2 Department of Public Health Dentistry, B. P. Koirala Institute of Health Sciences, Dharan, Nepal

**Keywords:** Community oral health programme, Dental anxiety, Pain perception, Periodontal probing

## Abstract

**Background:**

Periodontal probing is one of the basic clinical oral examination procedures. It is carried out to assess the severity of gingival and periodontal disease. The experience of pain during probing may discourage patients. So, this study was conducted to estimate the pain perception and dental anxiety experienced during periodontal probing in patients visiting the community oral health programmes of B. P. Koirala Institute of Health Sciences (BPKIHS).

**Methods:**

A cross-sectional study was conducted among 100 participants of community oral health programmes of BPKIHS. Demographic profile, WHO modified Community Periodontal Index (CPI) 2013, Pain perception via Visual Analogue Scale (VAS Scores) and Short Version of Spielberger State-Trait Anxiety Inventory (STAI) Self-evaluation Questionnaire (Y-6 item) were assessed. Mean ± SD and Spearman correlation for pain and anxiety were computed.

**Results:**

Only 10% of the study participants had healthy gingiva and 12% had periodontal pockets. Pain perception and dental anxiety was present in the participants. The participants experienced very little pain (6.75 ± 10.65) during periodontal probing. The overall anxiety score was 13.37 ± 1.81. There was a very weak correlation between the VAS Scores and the anxiety scores of the participants.

**Conclusion:**

This study concludes that pain perception and anxiety are low during periodontal probing. There was no correlation between bleeding on probing with pain and anxiety among the people visiting community oral health programmes of BPKIHS.

## Background

International Association for the study of Pain (IASP) defines pain as “an unpleasant sensory and emotional experience associated with actual or potential tissue damage, or described in terms of such damage” [1]. Pain cannot simply be determined by the intensity of nociceptive stimulation [2]. Pain not only is a physiological experience; rather includes cognitive and emotional construct [3]. Periodontal probe is a commonly used instrument to assess periodontal conditions and the severity of periodontal lesions [4, 5]. However, patient discomfort and pain associated with the insertion of a periodontal probe into the periodontal pocket are common clinical events [6].

Periodontal probing is used to measure clinical parameters like bleeding on probing, probing depth, clinical attachment level (CAL) and so on. This gives us an idea regarding the disease severity of periodontal structure.

The intensity of pain or discomfort has been perceived by practitioners to differ dramatically between patients [6]. The experiences of pain during probing and scaling may also discourage patients who do not have periodontitis. The individual characteristics such as age, smoking and oral health status effects patients’ pain perception and dental anxiety. An unpleasant dental experience has a strong impact on dental anxiety. Extreme pain experience after dental work is amongst the most common distressing life experiences, and has seen to trigger psychological trauma and a persistent fear of the dentist among patients [7].

Oral health education via different media changes attitude and practice as well as improves oral hygiene habits, oral health awareness and knowledge level [8]. Oral health education and promotion should be done to overcome the dental fear and anxiety [9]. The prevalence of deep periodontal pocket is around 31% in Nepal [10]. There is a paucity of research conducted to assess the pain perception and dental anxiety during periodontal probing and no research has been conducted in Nepal addressing the pain perception of the patient in this context. With an assumption that the pain perception and dental anxiety increases with periodontal probing, this study aims to assess the pain perception and dental anxiety during periodontal probing in patients visiting the community oral health programmes of B. P. Koirala Institute of Health Sciences (BPKIHS), Dharan, Nepal.

## Methods

**Fig. 1:**
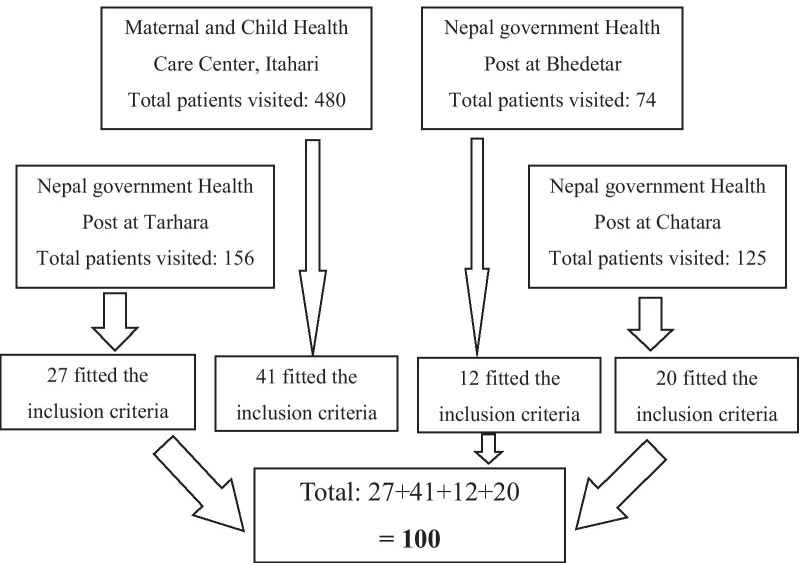
Flow diagram of the participants

A cross-sectional study was conducted among 100 patients visiting community oral health programmes of BPKIHS. The study was conducted from February 2018 to July 2018. Ethical approval for the study was obtained from the Institutional Review Committee, BPKIHS, Dharan (Ref. No: IRC/1033/017). The study adhered to STROBE guidelines. The community oral health programmes of BPKIHS is regularly conducted on weekly basis at Maternal and Child Health Care Center, Itahari, Nepal government Health Post at Bhedetar, Chatara and Tarhara. Adult Patients (18–75 years of age) who visited the Community Oral Health Programs of BPKIHS were included in the study (Fig. 1). We excluded the patients requiring prophylactic antibiotics before probing, patients suffering from mental disorders or with chronic pain problems, patients having previous experience of painful dental visit, patients suffering from coagulation/ bleeding disorders, pregnant or lactating mothers, patients under antidepressant and analgesic medications, patients having acute periodontal pain (with abscess, pulpitis or acute infections). This study considered (95% CI) to estimate the sample size. For this purpose, mean ± SD (19.1 ± 9.6) value was taken from the study [11] done by Canakci and Canakci. A convenience sampling method was used. All patients who visited the community oral health programmes and those satisfying inclusion and exclusion criteria were enrolled into the study.

### Survey instruments

- Questionnaire for accessing oral hygiene practices and demographic profile: The questionnaires were translated in Nepali followed by face and content validation by expert faculties.
- Visual Analogue Scale (VAS) [12]: It is most common method used in epidemiological studies to access pain perception. It is simple, reliable and most accepted method. The scale was anchored by “no pain” (score 0) and “pain as bad as it could be” or worst imaginable pain” (score of 100 [100-mm scale])
- Short Version of Spielberger State-Trait Anxiety Inventory (STAI) Self-evaluation Questionnaire (Y-6 item) [13]: It is a six item questionnaire that rate the anxiety on a likert-scale as 1: not at all anxious, 2: somewhat anxious, 3: moderately anxious and 4: very much anxious.
- Modified Community Periodontal Index as that of WHO Oral Health Survey Methods 2013 [14]: It scores the presence and absence of gingival bleeding and periodontal pocket of individual tooth.

*Procedures and schedules* Informed Consent was taken from all the patients. All the participants were examined by a trained and calibrated examiner, seated in a chair, under the natural light with a plane mouth mirror and WHO periodontal probe. Oral examination was followed by recording of the Modified Community Periodontal Index. Pain perception was assessed using Visual Analogue Scale for each quadrant. All the teeth in a quadrant were probed and participants were asked to rate their pain experience on VAS. Subsequently, the participants were asked to encircle the scores of the Short Version of Spielberger State-Trait Anxiety Inventory (STAI) Self-evaluation Questionnaire (Y-6 item) for assessing their anxiety experienced during periodontal probing. People diagnosed with dental caries, periodontal diseases and any other oral lesions were treated as per feasibility and resources available at the community field programs (like Atraumatic restorative Treatment and Extractions). Others were referred to the Department of Public Health Dentistry, College of Dental Surgery, B. P. Koirala Institute of Health Sciences, Dharan for needful treatment. A pilot examination was conducted to validate the entire questionnaire (pro forma) that was translated in Nepali version. The translation was done as per WHO oral health survey methods 2013 guidelines. The pilot study was conducted among 10 participants prior to the main study and these participants were not included in the main study. The Cronbach alpha value for the anxiety questionnaires and intra-class correlation coefficient for VAS Scores were found to be 0.76 (acceptable reliability) and 0.81 (good reliability) respectively.

#### Statistical analysis

After completion of the survey, data obtained were entered in Microsoft Excel Sheet 2007 and statistical analysis was done in Statistical Package for Social Sciences (SPSS) version 11.5 (SPSS, Inc., Chicago, IL, USA) software. Descriptive statistics including the mean and standard deviations were computed for VAS Score, anxiety score and CPI Score. Inferential Statistics: included; correlation between mean VAS Scores for the maxillary and mandibular arch obtained using Spearman Correlation Coefficient; and Mean VAS score and anxiety score compared based on gender using Kruskal–Wallis test.

## Results

Out of 100 participants, majorities were male (57%). Almost all (98%) of the participants were found brushing their teeth. About 80% were seen to brush for 2 to 5 min (Table 1).

**Table 1:** Demographic characteristics

Demographic characteristics	Percentage
*Gender*	
Male	57
Female	43
*Marital status*	
Married	51
Unmarried	49
*Socioeconomic status*	
Lower class	37
Middle class	63
Upper class	0
*Time taken for brushing*	
1–2 min	14
2–5 min	80
More than 5 min	6
*Type of tooth brush*	
Hard	13
Medium	5
Soft	19
Don’t know	62
*Type of toothpaste*	
Fluoridated	39
Non-fluoridated/don’t know	61

Only 10% of the study populations had healthy gingival. Twelve participants were found to have periodontal pockets of which one had a deep pocket of 6 mm or more (Table 2).

**Table 2:** Community Periodontal Index (WHO modified 2013)

Gingival status			Periodontal status		
	Scores	Percentage		Scores	Percentage
Absence of condition	0	10	Absence of condition	0	41
Presence of condition	1	72	Pocket 4–5 mm	1	11
Teeth excluded	9	0	Pocket 6 mm or, more	2	1
Tooth not present	X	47	Tooth excluded	9	0

Overall mean VAS score of the study participant was 6.75 ± 10.65 where males and females had similar VAS Scores (6.97 ± 11.53 and 6.46 ± 9.48 respectively). The difference in the VAS score among males and females was not significant (*p* = 0.780) (Table 3).

**Table 3:** Mean ± SD visual analogue scale score

Quadrant	Mean ± SD		
	Male	Female	Total
1	4.88 ± 8.79	3.74 ± 7.83	4.39 ± 8.35
2	5.98 ± 11.04	4.16 ± 9.47	5.20 ± 10.38
3	5.43 ± 9.01	10.60 ± 15.12	9.46 ± 14.46
4	8.60 ± 13.98	7.35 ± 12.27	7.98 ± 15.05
Overall	6.97 ± 11.53	6.46 ± 9.48	6.75 ± 10.65

*SD* Standard deviation

The mean age of male participants was 29.7 ± 11.8 years and that of female participants was 31.9 ± 10.6 years. All the participants were further divided into two categories based on age (less than 40 years and more than 40 years) to see the pain difference in young and old age groups. There was a statistically significant difference in VAS scores between both the age groups with old age participants having higher pain perception (*p* = 0.038). There was no statistically significant difference in the anxiety scores among both the age groups (*p* = 0.669) (Table 4).

**Table 4:** Comparison of VAS score and anxiety score between age groups

	Age group	n	Mean ± SD	Mean rank	*P*-value
Anxiety	18–40 years	85	13.54 ± 1.72	51.01	0.669
	41–75 years	15	13.07 ± 2.28	47.63	
VAS score	18–40 years	85	6.00 ± 9.50	48.08	0.038*
	41–75 years	15	11.05 ± 15.41	64.23	

*SD* Standard deviation

*Kruskal–Wallis test

There was a strong and statistically significant correlation of VAS scores between maxilla and mandible (*p* < 0.001) Table 5.

**Table 5:** Correlation between VAS Scores between maxilla and mandible

	Median	Mean	SD	Correlation coefficient	*P*-value
Maxilla	0.00	4.79	8.70	0.643	< 0.001*
Mandible	0.50	8.72	13.75		
Overall	2.50	6.75	10.65		

*SD* Standard deviation

*Spearman correlation coefficient

The dental anxiety was also similar in both males (13.60 ± 2.03) and females (13.30 ± 1.48) with overall anxiety score 13.37 ± 1.81 (Table 6).

**Table 6:** Mean ± SD of anxiety scores

	Mean ± SD	*P*-value
Male	13.60 ± 2.03	0.408*
Female	13.30 ± 1.48	
Overall	13.47 ± 1.81	

*SD* Standard deviation

*Kruskal–Wallis test

There was a weak significant correlation between VAS score and bleeding on probing whereas weak or no correlation between anxiety and bleeding on probing (Table 7).

**Table 7:** Correlation between bleeding on probing with pain and anxiety

Correlation		Anxiety	Pain (VAS score)
Bleeding on probing	Correlation coefficient*	0.033	0.350**
	Sig. (2-tailed)	0.742	0.000
N		100	100

*Spearman correlation coefficient

**Correlation is significant at the 0.01 level (2-tailed)

There was a very weak correlation between the VAS Scores and the anxiety scores of the patient (Table 8).

**Table 8:** Correlation between VAS score and anxiety scores

	Anxiety	VAS score
Correlation coefficient	1.000	0.103*
Sig. (2-tailed)		0.310
N	100	100

*Spearman correlation coefficient

## Discussion

This study provided information about pain perception and dental anxiety during periodontal probing. Oral examination was conducted in oral health outreach programmes, on a chair in a sitting position under natural light. The findings revealed, 72% of the participants had pathological gingival conditions but very few had periodontal pocket (12%).

Pain perception of the patient cannot be directly assessed by the dentists as communication skills, individual psychological status and, social and cultural backgrounds of the patient affect the expression of pain experienced. In this study, the participants experienced very less pain (6.75 ± 10.65) during periodontal probing but the VAS scores were highly variable (0 to 59.5). Similar results were obtained in other studies [1, 2, 7]. This might be attributed to the reason that pain measurement is subjective and individual, and the assessment and screening are more difficult because of its physical and psychological properties [15]. Additionally, pain perception is influenced by the patients’ systemic conditions, oral pathological status, and patients reporting with the complaint of pain [6, 15].

Pain perception of female participants was similar to the male participants (6.46 ± 9.48 and 6.97 ± 11.53 respectively), and the difference was not statistically significant (*p* = 0.780). This was similar to the study conducted by Canakci and Canakci [11] but different than the results of Faisal et al. [16]. In general, the clinical impression is that elderly people are usually more tolerant of pain. Nociceptors are lost due to aging [17]. In contrast, this study showed a higher VAS score in the elderly (11.05 ± 15.41) compared to young participants (6.00 ± 9.50). This might be due to the reason that pain varies subjectively and is also dependent upon many underlying causes that might be unnoticed clinically. There was a significantly significant correlation between the VAS score of maxilla and mandible. The correlation between bleeding and VAS score was also significant (*p* < 0.001). Bleeding on probing indicated the inflammatory condition of the gingiva that raises the possibility of increased pain perception [5].

Female patients are more anxious than male patients. It may be due to the difference in pain threshold between genders. In contrast, this study revealed that the dental anxiety was also similar in both males (13.60 ± 2.03) and females (13.30 ± 1.48) with overall anxiety score 13.37 ± 1.81. The anxiety of female participants was less compared to the male participants (13.30 ± 1.48 and 13.60 ± 2.03 respectively), but the difference was not statistically significant (*p* = 0.408). The insignificant higher anxiety score in male participants might be attributed to difference in sample participants (male = 57; female = 43). The higher mean age of the female participants (31.9 ± 10.6) might have also attributed to insignificant less anxiety score among female. It has been seen that older individuals experience lesser anxiety than their younger counterparts due to general decline in anxiety and many more exposure to diseases and their treatments [18]. This was similar to a study conducted by Shaikh and Kamal [19] and Ghazaleh et al. [20] but different than the results of Faisal et al. [16].

Anxiety is thought to increase pain perception and vice versa [21]. Female is supposed to have more fear compared to the male [21]. In contrast, in this study the anxiety scores for males and females were comparable. There was a very weak correlation between anxiety score and bleeding on probing and anxiety and pain perception. This might be due to the fact that anxiety has an influence on expected pain, but not on the experienced pain [22].

The sampling covered a large population area including four districts of eastern Nepal. This study is the first of its kind done in the population. Hence, it is an added asset for the dentist to have an insight into the pain perception and dental anxiety during periodontal probing and further plan the approach for patient management.

The sampling technique used was convenience sampling that limits the generalizability of the study and gives a scope for selection bias. Moreover, the probing was performed by the WHO probe that increases the chance for subjective variation of force applied during probing. A digital probe would have been better to maintain the constant force of probing. The other limitation being chances of variation in pain response by the patients as full mouth probing was done and there were partially edentulous patients. Measuring anxiety accurately is extremely difficult and therefore it may alter research outcomes. As pain and anxiety are subjective measures, it is difficult to quantify them. A participant may express pain and anxiety to one aspect of examination but not in another. Hence, experimental studies with digital monitoring of the probing force will further elaborate regarding the correlation between pain and anxiety.

## Conclusion

This study found low pain perception and anxiety during periodontal probing and there was a very low correlation between bleeding on probing with pain and anxiety among the people visiting community oral health programmes of BPKIHS.

## Data Availability

The data supporting the findings of this article are available from the corresponding author.

## Abbreviations

BPKIHS: B. P. Koirala Institute of Health Sciences
WHO: World Health Organization
ART: Atraumatic restorative treatment
